# Stress of conscience of COVID-19 among perianaesthesia nurses having worked in a COVID-ICU during the coronavirus pandemic: an international perspective

**DOI:** 10.1186/s12912-022-00862-w

**Published:** 2022-04-07

**Authors:** Ulrica Nilsson, Jan Odom-Forren, Mette Ring, Hanneke van Kooten, Joni M. Brady

**Affiliations:** 1 Department of Neurobiology, Care Sciences, and Society, Karolinska Institute, and Perioperative Medicine and Intensive Care, Karolinska University Hospital, Stockholm, Sweden; 2 College of Nursing, University of Kentucky, Lexington, KY USA; 3 Clinic of Anaesthesiology, Aalborg University Hospital, North Denmark Region, Aalborg, Denmark; 4 Martini Hospital, Groningen, Netherlands; 5 International Collaboration of PeriAnaesthesia Nurses, Alexandria, USA

**Keywords:** Stress of conscience, COVID-ICU, Perianaesthesia nurses, Usual workplace

## Abstract

**Background:**

Several studies have reported that working in a COVID-ICU impacted nurses’ mental well-being. Yet little is known about how perianaesthesia nurses who have been working in a COVID-ICU perceived their stress of conscience. The aim of this study was to: (1) describe and compare stress related to troubled conscience among perianaesthesia nurses in three countries who have been working in a COVID-ICU during the pandemic, (2) compare their levels of troubled conscience between working in a COVID-ICU and their usual workplace, and (3) compare nurses that usually work in an ICU department with nurses who usually work outside of the ICU.

**Methods:**

A descriptive, international cross‐sectional online survey including the Stress of Conscience Questionnaire (SCQ) was distributed between organizational member countries of the International Collaboration of PeriAnaesthesia Nurses.

**Results:**

A total of 246 nurses from three countries participated. Significant differences were found in stress of conscience when working in the Covid-ICU between Sweden 31.8 (8.6), Denmark 23.1 (8.6), and Netherlands 16.4 (6.5) *p* < 0.001. Significant differences were also found between nurses working in a COVID-ICU in contrast with their usual workplace: 23.1(5.6) versus 17.7(5.3), *p* < 0.001. The most stressful aspect of conscience reported was that work in the COVID-ICU was so demanding, nurses did not have sufficient energy to be involved with their family as much as they desired. No statistical differences were found between nurses that usually work in an ICU department with nurses who usually work outside of the ICU.

**Conclusion:**

The COVID-19 pandemic has negatively impacted stress of conscience among nurses working in the COVID-ICU. Swedish nurses were found to be more significantly impacted. This could be related to low numbers of existing ICU beds and ICU nurses prior to the pandemic necessitating a longer time required for working in a COVID-ICU. Stress of conscience also increased when working in the Covid-ICU compared to working in the usual workplace, and the most stressing aspect reported was that COVID-ICU work was so demanding that nurses did not have the energy to devote themselves to their family as they would have liked.

## Background

Currently, many nurses cope with high stress levels in their work environment. When time constraints are present, each nurse must prioritize which nursing actions to provide and which to omit (called missed nursing care), which can result in decreased patient safety [1, 2]. The nurse may experience a feeling of guilt and inadequacy when unable to perform the standard of nursing care required [3]. Missed nursing care can lead to moral dilemmas that influence the conscience [3, 4]. 

Conscience can be described as a warning system alert when our personal and professional values, beliefs, ethics, and standards become threatened by issues or circumstances encountered [5]. While professional conscience has the potential to guide and inform the provision of high quality care, it can also lead to significant challenges for the nurse [5]. Nurses experience stress from their conscience when impeded from taking the “right” action or being obliged to act in a way that they believe is not good enough, or even wrong [3, 5, 6]. Troubled conscience or stress of conscience [1, 5, 6] is closely linked with stress and strain, which correlates to circumstances in which a nurse does not act in accordance with their own conscience [6].

Nurses find themselves caught between an ideal image of health care and the reality of their care delivery experiences, resulting in a troubled conscience from the inability to do more [3, 6, 7]. Stress of conscience is a product of the frequency of stressful situations and the perceived degree of troubled conscience as rated by nurses themselves [3]. Nurses’ troubled conscience can affect their job satisfaction [8] and personal lives [6, 9], and even cause burnout [7]. Furthermore, nurses suffering from a troubled conscience may need to distance themselves in order to cope with the work situation, which may impair their caring relationships with patients [10].

Provision of care for COVID-19 infected persons in a COVID Intensive Care Unit (COVID-ICU) can be very demanding and ethically challenging [11]. Observers in a COVID-ICU have reported an increase in nursing workload due to a higher than usual nurse-to-patient ratio and the intensification of nursing activities. The intensive care interventions required for a person suffering from COVID-19 as compared with a typical ICU patient has been recognized as more complex and demanding. This is because COVID-19 patients develop more severe complications, such as acute respiratory failure, septic shock, and renal insufficiency [12].

Several studies have reported that working in a COVID-ICU highly impacts mental well-being [13–19] and experiences of moral conflicts [20]. Yet, little is known about how perianaesthesia nurses who have been working in a COVID-ICU perceived their conscience. Given the sustained demand for intensive care nursing skills needed to manage COVID-19 patients throughout the pandemic, perianaesthesia nurses may be “running on empty” and suffering from retrospective guilt [21]. This feeling of guilt may give rise to stress related to a troubled conscience.

Making ethical choices significantly impacts nurses' care performance [4, 20]. Whether these ethical choices and demands differ when working in a COVID-ICU as compared to the perianaesthesia nurses’ usual workplace is unknown. Further, does the nurse’s stress of conscience differ if their usual workplace is a designated ICU, or not, because the environment and most care delivery equipment is the same in a COVID-ICU. It has been reported that non-specialized intensive care nurses feel poorly trained and prepared for working in a COVID-ICU treating severely ill patients[13].

Therefore, the aim of this international study is to: (1) describe and compare stress related to troubled conscience among perianaesthesia nurses in three countries who have been working in a Covid-ICU during the Coronavirus pandemic, (2) compare their levels of troubled conscience between working in a Covid-ICU compared to their usual workplace, and (3) compare nurses that usually work in an ICU department with nurses who usually work outside of the ICU.

## Material and methods

### Design

A descriptive, international cross‐sectional survey including the Stress of Conscience Questionnaire (SCQ).

### Participants

A convenience sampling approach was used in this study. A consecutive sample of perianaesthesia nurses were invited to participate from the International Collaboration of PeriAnaesthesia Nurses’ (ICPAN) organizational member countries comprising its Global Advisory Council (GAC). Only three of the 11 participating member organizations (Netherlands/BRV, Denmark/FSAIO, Sweden/ANIVA) were included in the results because the response rate from eight other participating countries was too low.

### Questionnaire

The Stress of Conscience Questionnaire (SCQ) [3] estimates stress related to troubled conscience. The SCQ instrument consists of 9 items, distributed into two parts: A and B. Part A addresses how often a certain stressful care situation occurs in the participant’s workplace, whereas Part B measures the degree of troubled conscience this specific situation evokes. The responses on Part A are rated on a 6‐point Likert‐type scale form ‘Never’ to ‘Every day’ (Table 1). For Part B, the response scale is a visual analogue scale ranging from 0 = ‘No, not at all’ to ‘5 = Yes, it gives me a very troubled conscience’. A stress of conscience index is calculated by multiplying scores of each A-question with the scores of its corresponding B-question. A sum score of all the items is calculated, with higher values indicating higher stress of conscience (0–350). Items 1, 2, 4, 5, and 9 are a subscale defines Internal Demands; Items 3, 6, 7, and 8 are defined External Demands and Restrictions [3]. More recent work has determined that the SCQ is more unidimensional than two-dimensional [22]. The overall scores can be meaningful to compare. However, we compared both internal, external, and overall scores because further exploration of the SCQ is required. Cronbach’s alpha has been found to be 0.83 for the nine-item total [3].

**Table 1:** Example of modification of the SCQ questions

Original SCQ	COVID-ICU	Usual workplace
How often do you lack time to provide the care the patient needs?	How often did you lack time to provide the care the patient needs in the COVID -ICU?	How often do you lack time to provide the care the patient needs in your usual workplace?

### Modification of the SCQ

The Stress of Conscience Questionnaire has been modified to suit the purpose of this study to measure both stressful COVID-ICU care situations encountered and how often that same situation has occurred in the nurse’s usual workplace (Table 1).

The response scale was also modified to include a new item: every hour (Table 2).

**Table 2:** Modification of the response scale

Original SCQ	COVID-ICU	Usual workplace
1Never’ 2Less than once/6 months’ 3More than once/6 months’ 4Every month’ 5Every week’ 6Every day’	1Never’ 2Less than once/6 months’ 3More than once/6 months’ 4Every month’ 5Every week’ 6Every day’ 7Every hour’	1Never’ 2Less than once/6 months’ 3More than once/6 months’ 4Every month’ 5Every week’ 6Every day’ 7Every hour’

Permission to translate and modify the SCQ was obtained from one of the originators of the questionnaire, Dr. Gunilla Strandberg (personal communication, Sept 2020). An expert nurse panel including members of the ICPAN board (*n* = 7), GAC representatives (*n* = 11), and one researcher reviewed the modified SCQ. The review resulted in some linguistic modifications of the items.

### Rationale for questionnaire modification

It is important to analyze if there are any differences in how the nurses experienced their care encounters and stress of conscience when working in a COVID-ICU compared to their usual workplace. Colleagues working in a COVID-ICU have reported that they could not provide all of the care that they should have, i.e., missed care, and expressed alarm over the frequency of occurrence [13]. Therefore, due to the higher acuity of care, we included the item “Every hour”. We also changed the tense for the COVID -ICU questions to include past and present, because many nurses had returned to their usual workplace while others may have been reassigned to the ICU setting after encountering waves of COVID-19 admission cases. The tense for the question about stress of conscience when working in a COVID-ICU was changed to both past and present resulting from a second and third wave of the global pandemic. For example: How often have you/ did you lack time to provide the care the patient needs in the COVID-ICU?

### Content validity testing

After a pilot test with one perianaesthesia nurse (a Danish nurse anaesthetist who had been working in a COVID-ICU for 3 months) question number 9 was changed from ‘Did you ever lower your aspirations to provide good care in the COVID-ICU?’ to ‘Did you ever lower your expectations to provide good care in the COVID-ICU?’ (i.e., aspirations changed to expectations for clarity in translation). A Swedish nurse having 1 month of experience in a COVID-ICU also tested the SCQ with no further changes made. An English online version of the SCQ was then pilot tested by the research team and ICPAN board members (*n* = 12). This led to minor changes that focused on survey demographic variables.

### Language translation

The modified version of The Stress of Conscience Questionnaire and the demographic questions were translated into Dutch, Danish and Swedish. The translations were performed by members of the ICPAN board and current or former GAC members who spoke fluent English in addition to their national language. The translated surveys were compared to the English version and discussed among the members to promote content consistency. This discussion resulted in some minor linguistic modifications of the items.

### Procedure

The online survey was distributed from KI Survey&Report, which is built on the safe and secure platform Microsoft ASP.NET at Karolinska Institutet in Sweden. The members of the ICPAN GAC were responsible for sending the online survey to the perianaesthesia nurses in their respective country. The online survey invitation was published on each GAC organization’s social media account with one additional reminder disseminated.

### Data analysis

Data are presented with descriptive statistics: frequency, percent, mean, median and standard deviation (SD). Research question 1 and 2 were analyzed with Kruskal Wallis ANOVA followed by Mann–Whitney U-test and a Bonferroni correction in order to test differences between the three countries in demographic variables, overall scores of Stress of Conscience, the internal demand and external demand factors, as well as the stress of conscience index. Research question 3 that referenced nurses whose primary workplace was the ICU included only the Swedish and Danish samples. These nurses were dichotomized into usual workplace ICU and usual workplace outside ICU (Anesthesia, Recovery room and Day surgery) and analyzed with Mann Whitney U test. The sample from the Netherlands was not included because their organization does not include nurses working in ICU. Duration of time working in the COVID-ICU was dichotomized into ICU ≤ 20 weeks vs > 21 weeks guided by the average duration of time i.e., Cronbach’s alpha (α) was used to determine the internal consistency of the questionnaire. The *p*-value < 0.01 was considered statistically significant in all analyses.

## Results

A total of 246 nurses participated, of whom the majority were from Netherlands (*n* = 104; 42.3%) followed by Denmark (*n* = 72; 29.3%) and Sweden (*n* = 70; 28.5%). The mean age was 45.5 years (SD 10.29) with work experience as a nurse of 21.5 years (SD 10.9) and duration of time working in the COVID-ICU of 20.8 weeks (SD 14.3), Table 3. The majority of the nurses self-identified as female 211 (87.2%). There were no significant differences found between female and male respondents in the mean age 45.8 (SD 9.8) vs 43.1 (SD 11.7), work experience as a nurse 22.6 (SD 10.5 vs 17.7 (SD 13.1) years, or duration of time working in the Covid-ICU 20.6 (SD 14.2) vs 21.5 (SD 14.8) weeks. There were significant differences between the countries in academic degree level, with lower academic preparation levels reported by the Danish nurses as compared to the Swedish and Dutch nurses, *p* < 0.001. No Dutch nurse respondents reported having usually worked in an ICU as compared to Denmark (*n* = 42; 59.2%) and Sweden (*n* = 53; 76.8%), *p* < 0.001.

**Table 3:** Demographics

	**Total** ***n* = 244**	**Sweden** ***n* = 69**	**Netherland** ***n* = 104**	**Denmark** ***n* = 71**	***p*-value**
Age, Mean (SD)	45.5 (10.2)	43.5 (8.9)	46.0 (11.0)	46.9 (10.2)	n.a
Gender					n.a
Female	211 (87.2%)	61(88.4%)	84(80.8%)	66(93.0%)	
male, n (%)	31 (12.8%)	8(11.6%)	20 (19.2%)	5 (7.0%)	
Academic degree, n (%)					< 0.001*
Non	37(15.2%)	2(%)	9(%)	26(%)	
Bachelor	84(%)	21(%)	27(%)	36(%)	
Master	18(%)	45(%)	64(%)	9(%)	
PhD	5(%)	1(%)	4(%)	-	
Usual workplace, n (%)					< 0.001**
ICU	95(38.9%)	53(76.8%)	27(26.0%)	42(59.2%)	
Anaesthesia	64(26.2%)	15(21.7%)	54(51.9%)	22(31.0%)	
Recovery room/ PACU	60(24.6%)	1(0.1%)	23(22.1%)	5(7.0%)	
Day Surgery	25(10.2%)			2(3.0%)	
Work experience as a nurse, years, Mean (SD)	21.5 (10.9)	18.4 (9.2)	24.1 (11.8)	20.8 (10.4)	n.s
Duration of time working in the Covid-ICU (weeks), Mean (SD)	20.8 (14.3)	30.0(12.3)	14.2 (11.6)	21.6 (14.8)	< 0.001***
Have received a Covid positive test or immunity test, n (%)	30 (12.3%)	10(/14.5%)	16 (15.4%)	4(5.6%)	N.S

*PACU* postanesthesia care unit

Bonferroni correction: *Denmark vs Netherlands and Sweden; ** Netherlands vs Denmark vs Sweden; *** between all three countries

### Comparisons of stress of conscience between perianaesthesia nurses in three countries who have been working in a Covid-ICU

Cronbach’s alpha (α) for: (1) the overall score of the SCQ in the total sample, when working in the COVID-ICU, was 0.84, (2) the factor Internal demands when working in the COVID-ICU was 0.74, and (3) Factor II, External demands and restrictions when working in the COVID-ICU was 0.68 for the total sample.

There were significant differences between all three countries in the distribution of overall scores of Stress of Conscience when working in the COVID-ICU, as well in Internal and External demands, *p* < 0.001 (Table 4). No statistical differences were found between type of nurses and overall scores of Stress of Conscience when working in the COVID-ICU, and Internal demands and External demands neither for working in the COVID-ICU or in the usual workplace. Statistically significant differences were found between Overall scores of Stress of Conscience working in the Covid-ICU between nurses working in that setting for ≤ 20 weeks vs > 21 weeks, 20.7 (9.7) vs 25.5(9.8), *p* < 0.001. Statistical differences were also found between the factors Internal demands, 11.1(6.1) vs 14.4(6.2), *p* < 0.001, but not in the factor External demands. There were significant differences between the comparison countries in 8/9 stress of conscience index measures. The Swedish nurses rated higher values in all 8 stress of conscience indices, with the highest value reported for: “Your work in the Covid-ICU was ever so demanding that you did not have the energy to devote yourself to your family as you would have liked?” resulting in: 20.3(SD 11.5) of Swedish nurses vs Danish nurses 12.5 (SD 10.7) and Dutch nurses 6.8 (SD 8.9), *p* < 0.001. The stress of conscience index for this question was not significant between the nurses (Table 5).

**Table 4:** Differences between Swedish, Dutch, and Danish nurses in overall scores and factors of Stress of Conscience when working in the Covid ICU and when working in the usual workplace

	**Total** ***n* = 244** **Mean (SD)** **range**	**Sweden** ***n* = 69** **Mean (SD)** **range**	**Netherland** ***n* = 104** **Mean (SD)** **range**	**Denmark** **Mean (SD)** **range**	***p*-value**
Factor I, Internal demands when working in the Covid ICU	12.6(6.3) 5–30	18.4 (5.5) 5–30	8.8(4.0) 5–21	12.3(5.6) 5–7	< 0.001*
Factor II, External demands and restrictions when working in the Covid ICU	11.7(5.3) 4–27	15.2 (4.7) 4–27	9.1(4.3) 4–23	11.9 (5.0) 4–20	< 0.001*
Overall score, Stress of Conscience when working in the Covid ICU	23.1(5.6) 9–51	31.8 (8.6) 9–51	16.4 (6.5) 9–37	23.1 (8.6) 9–42	< 0.001*
Factor I, Internal demands when working in the usual workplace	9.5(3.0) 5–17	10.4 (2.9) 5–17	8.8(2.9) 5–17	9.7(3.1) 5–17	< 0.001**
Factor II, External demands and restrictions when working in the usual workplace	8.2(2.9) 4–17	9.2(2.7)	7.6(2.9) 4–17	8.0(2.9) 4–15	< 0.001**
Overall score, Stress of Conscience when working in the usual workplace	17.7(5.3) 9–31	19.6(4.7) 0–30	16.4(17.1) 9–30	17.8(5.2) 9–31	< 0.001**

Bonferroni correction: * Between all three countries; **Sweden vs Netherland

**Table 5:** Differences between Dutch, Danish and Swedish nurses in stress of conscience index (range 0–35)

	**Netherland** ***n* = 104** **Mean (SD)** **Range**	**Denmark** ***n* = 71** **Mean (SD)** **Range**	**Sweden** **n = 69** **Mean (SD)** **Range**	***p*-value**
1.Lack time to provide the care the patient needed in the Covid-ICU? (internal demands and external demands/restrictions)	1.4(2.8) 0–12	3.0(4.8) 0–18	9.6 (5–7) 0–21	< 0.001**
2.Forced to provide care that felt wrong in the Covid-ICU (internal demands)	2.2 (4.4) 0–20	7.4(9.5) 0–35	15.5 (9.9) 0–35	< 0.001*
3.Deal with incompatible demands in your work in the Covid-ICU (external demands/restrictions)	2.0(4.9) 0–28	4.4 (8.1) 0–35	15.5 (11.4) 0–35	< 0.001***
4.See patients being insulted and/or injured in the Covid-ICU (internal demands)	0.3(1.0) 0–5	4.4(8.1) 0–35	7.1(10.0) 0–35	< 0.001***
5.Find yourself avoiding patients or family members who need help or support in the Covid-ICU (internal demands)	0.8(2.7) 0–21	3.2(7.8) 0–35	5.7(8.2) 0–35	< 0.001****
6.Your private life was ever so demanding that you did not have the energy to devote yourself to your work in the Covid-ICU as you would have liked (external demands/restrictions)	3.1(6.5) 0–35	3.4(6.4) 0–28	2.5(6.5) 0–35	n.s
7.Your work in the Covid-ICU was ever so demanding that you did not have the energy to devote yourself to your family as you would have liked (external demands/restrictions)	6.8(8.9) 0–35	12.5(10.7) 0–35	20.3(11.5) 0–35	< 0.001*
8.Feel that you cannot live up to others expectations of your work in the Covid-ICU (external demands/restrictions)	6.4/10.8) 0–35	7.0(10.7) 0–35	12.4(12.3) 0–35	< 0.001**
9.Lower your ambitions to provide good care in the Covid-ICU (internal demands)	0.5(1.2) 0–5	2.2(2.0) 0–5	3.4(1.6) 0–5	< 0.001*

Bonferroni correction: * Between all three countries; **Sweden vs Netherland and Denmark: ***Netherland vs Sweden and Denmark; ****Sweden vs Netherland

### Comparisons in levels of troubled conscience between working in a Covid-ICU compared to their usual workplace

There were significant differences between all three countries in the distribution of overall scores of Stress of Conscience when working in the usual workplace and Internal and External demands, yet only between the Swedish nurses compared to the Dutch nurses, *p* < 0.001 (Table 4). Statistically significant differences were found between Overall scores of Stress of Conscience between working in the COVID-ICU vs working in the usual workplace, 23.1(5.6) vs 17.7(5.3), *p* < 0.001. Statistical differences were also found between the factors; Internal demands, 12.6(6.3) vs 9.5(3.0) and External demands, 11.7(5.3) vs 8.2(2.9) when comparing working in the COVID-ICU with working in the usual workplace, *p* < 0.001.

### Comparison between nurses that usually work in an ICU department with nurses who usually work outside of the ICU

There were no significant differences in mean age 44.3 (SD 10.0) vs 46.8 (SD 8.3) and work experiences as a nurse 18.7 (SD 9.9) vs 21.4 (SD 9.7) between the Danish and Swedish nurses who usually worked in an ICU as compared to the other nurses. The ICU nurses’ time working in the COVID-ICU was significantly longer 31.0 (SD 1.5) compared to the other nurse specialties 14.6 (SD 10.9). No statistical differences were found between type of nurses and overall scores of Stress of Conscience when working in the COVID-ICU and the factors of Internal demands and External demands. Statistical differences were found in overall scores of Stress of Conscience when working in the usual workplace, ICU nurses 19.4 (SD 5.1) vs Other nurse specialties 16.9 (SD 4.7), *p* < 0.01. The ICU nurses reported significantly higher Internal demands when working in the usual workplace 10.6 (SD 2.8) compared to Other nurse specialties 8.8 (SD 2.8), *p* < 0.001. No differences were found between the nurses in External demands and restrictions when working in the usual workplace.

## Discussion

This descriptive, international cross‐sectional study is the first study to describe and compare stress related to troubled conscience among perianaesthesia nurses from three countries who have been working in a COVID-ICU during the Coronavirus pandemic as compared to their usual workplace and compared to nurses who usually work in an ICU. The main findings were that overall SCQ scores and item scores were rated significantly higher when nurses were assigned to work in a COVID-ICU versus working in their usual workplace. Interestingly, there was a significant difference between all three countries when working in a COVID-19 unit. However, when working in the usual workplace, Swedish SCQ scores were significantly higher than the Netherlands, but not Denmark.

Even though Denmark and Sweden handled national management of the COVID-19 pandemic differently, those nurses have had similar responses to stressors from the pandemic: the reallocation of resources, lack of clear clinical guidelines and, most interestingly in terms of a stressed conscience, worry about not being able to provide patients appropriate treatment [23]. Lake et al [17] explored factors associated with nurses’ moral distress and found that pandemic patient care situations were the largest source of moral distress. In that study, ICU nurses had the highest rate of moral distress.

It is difficult to determine why the Swedish overall SCQ scores were higher than Denmark or the Netherlands when caring for COVID-19 patients or in the usual workplace. When looking at the 9-item SCQ, Sweden had the highest stress of conscience level on 8 of the singular items. We found that nurses who worked in a COVID-ICU for 21 weeks or more had significantly higher SCQ scores. The mean number of weeks working in the ICU in Sweden was 30 weeks compared to Denmark (21.6) and Netherlands (14.2). So, it is very possible that the length of work time in the COVID-ICU in Sweden contributed to an increased stress of conscience. Also, when looking at demographics, many of the nurses from Sweden and Denmark reported having worked mainly in the ICU, while the nurses from Netherlands were mainly working in anaesthesia, the postanaesthesia care unit (PACU), and Day Surgery units. We postulate that it is possible the Swedish critical care nurses are most aware of best practices in caring for ICU patients compared to the Netherlands who do not work in the ICU, causing more suffering from stress of conscience due to new circumstances encountered while working in a COVID-ICU. However, that does not explain why Denmark’s overall SCQ scores are significantly lower than Sweden, although higher than the Netherlands. Differences in the provision of supportive strategies for the nurses in the different counties may have been a factor. Clark et al [24] found that key supportive strategies include: ensuring that the staff receive time off to rest and recuperate; monitoring and support for the long-term mental health of staff; and ensuring that staff received recognition and gratitude for their service. Staff training needs are also of great importance [24]. Perhaps the Netherlands deployed a more well developed training process and provided ongoing emotional support for its nurses.

Nurses with < 10 years of practice experience participated in lower numbers, which may indicate some demographic barrier existed for taking the survey. We also know that perioperative nurses have reported fear of leaving the familiar for the unknown [25]. In this study we found that the most stressing aspect of conscience for nurses working in the COVID-ICU was related to the high job demands and the lack of energy to devote themselves to their family as they would have liked. However, work: family conflict is a well-known and crucial problem encountered in nursing due to demanding workplace conditions even before the pandemic. This balancing act between work and personal life can threaten nurses’ health, leading to emotional exhaustion [22]. That this aspect of conscience was experienced as most stressful is not surprising, and can be an explanation to earlier studies reporting that working in a COVID-ICU has a high impact on the mental well-being of nurses [13–19]. Another aspect of sense of conscience “forced to provide care that felt wrong in the Covid-ICU” was highly rated and aligns with other research such as the Sugg et al. [4] study about missed care among “COVID-nurses.” They found that the “COVID-nurses” struggled to support patients’ emotional wellbeing and mental health, and felt that they were unable to provide usual levels of support, reassurance, and interaction with patients. They also felt a lack of maintaining dignity and respect for patients’ values and beliefs [4].

Sweden experienced more COVID-19 deaths per capita, which may have contributed to the higher SCQ scores [26, 27]. It has been reported that inadequate workforce, having to triage patients due to lack of beds and/or equipment, being responsible for other staff members, and being asked to work in an area that was not in the respondents' expertise are factors associated with higher levels of stress when working in critical care settings during the early stages of the Corona pandemic [15]. Whether these factors differ between the countries is currently unknown. Yet, Bergman et al. found that the introduction to the COVID-19 ICU varied in both content and length and resulted in a feeling of unpreparedness among Swedish nurses [13]. Further, prior to the COVID-19 pandemic, Sweden had the lowest number of ICU beds per capita among the Nordic countries and rapidly scaled up its surge ICU capacity, enabling care for more individuals [27].

It is interesting to note that the Netherlands had similar scores regarding the work in a COVID-ICU versus a usual workplace because none of the participants reported that their usual workplace was the ICU. Initially, we believed that the Netherlands might have a higher level of stress of conscience because when working with COVID-19 patients, they were not in their usual workplace. This aligns with studies having reported that working in an area that is not in the respondents' expertise or not trained in ICU care are associated with a higher degree of stress, depression and anxiety [15, 28]. However, it is possible that because the ICU was not the usual workplace for the Dutch nurses they were given tasks versus usual nursing care, or possibly assigned to assist other experienced ICU nurses. It is also possible that the Dutch nurses were more clinically prepared and experienced a more suitable workforce, or that their limited time in the COVID-ICU (m = 14.2) contributed to the lower scores. When looking at COVID-19 hospitalizations and persons treated in ICU during the times our survey was open, Sweden and the Netherlands had similar population infection numbers with Denmark having the lowest numbers (ourworldindata.org). So those numbers do not explain the difference between Sweden’s SCQ scores with Denmark and the Netherlands either.

Of interest, Jokwiro et al [29] conducted a scoping review on the extent and nature of stress of conscience among healthcare workers. All 24 of the studies in the scoping review were conducted prior to COVID-19 pandemic. Yet, the SCQ scores were much higher than the scores in this study. For example, the highest mean score reported (0–225) was 63.6 in Registered Nurses (RNs) and nurse assistants who cared for older patients, and the lowest score reported was 24 in a mix of healthcare workers who cared for older, dementia patients [16]. The highest mean overall score in our study was 31.8 (0 – 350). We are not sure why the stress scores among the studies in the scoping review are higher than the scores in this study. It is possible that this finding is related to the differences in personnel and settings. For example, the persons having the most responsibility (the RNs) had higher stress levels than the nurse assistants. The important point is that healthcare workers experience stress of conscience, and we must find and implement consistent evidence-based strategies to mitigate stress and promote workforce wellness and resilience.

### Implications for practice and research

It is clear that nurses are suffering from stress of conscience during the pandemic partially due to: lack of resources; exhaustion from delivering complex care to COVID-19 infected patients; lack of preparation; poor leadership; repeated episodes of moral distress; and anxiety over the possibility of infection spread to themselves or their families [29–31]. Nurses have reported working because of a “sense of duty” [32]. More efforts to protect nurses physically, psychologically and socially are a health care system imperative [14]. Policy suggestions for mitigation include: allocation of necessary funding to provide essential protective equipment to all nurses; financial investments that improve nurse staffing; provision of psychological, physical, financial, and social support to nurses; and, ensuring a safe and positive work environment through legislation [19].

Other suggestions to decrease nurses’ stress include stable working conditions and better salaries [23]. Morensen et al [19] and Lake et al. [17] point out the importance of not only focusing on the mental health of frontline nurses, but that leaders at every level should provide clear and consistent communication to their teams. Effective communication from leaders can decrease moral distress [17, 33]. Nurses working during the pandemic have also pointed out that the hero narrative divests policymakers of any responsibility and instead places it on the individual nurse or facility. The media can play a role by covering the pandemic realistically and without using the hero narrative as the only lens [34]. The other narrative of nurses having responsibility for their own resilience has become a superficial response. By focusing on individual human responses, less attention is given to adequate organizational support that is a key component in creating positive working conditions [35]. Organizational resilience can be fostered by addressing the priorities listed above. Other important suggestions for the mitigation of nurses’ stress are consistent support to frontline nurses, recognition of exposure to COVID-19 as a work-related injury, and addressing the violence and stigmatization of healthcare workers that has occurred in some countries during the pandemic [32].

Further research on stress of conscience should be conducted. From the work of Jokwiro et al [36] we know that nurses were experiencing moral distress before the pandemic. The pandemic has further exacerbated that distress, resulting in nursing burnout and nurses leaving the profession. There are few interventional studies that have focused on stress of conscience and how to effectively decrease that stress. Randomized controlled trials and phenomenological investigation would add to the scientific knowledge around stress of conscience. Research could also address the predictors and causes of COVID-19 related workplace violence and stigmatization [19].

### Limitations

There are several limitations to this study. Our sample was a convenience sample of nurses from three countries who completed a self-survey. We do not know the differences between those who completed the surveys and those who did not, nor between those nurses in other countries who did not complete the surveys. We do not know the possible response rate because of the international aspect of this survey. There are variables we may not have collected that contributed to the stress of conscience. As the pandemic has continued to impact healthcare workers for an extended period, a more detailed survey in the future may provide further information on the impact to practicing nurses. However, a strength of the study is a compilation of data from three countries as nurses struggle with life in the pandemic era.

## Conclusion

In conclusion, stress of conscience is a product of the frequency of the stressful situation and its perceived degree of troubled conscience as rated by nurses themselves. The COVID-19 pandemic has had a negative impact on stress of conscience among nurses working in the COVID-ICU. However, the Swedish nurses were significantly more affected with higher levels of stress of conscience reported, which could be because Swedish nurses spent a longer time working in a COVID-ICU than the other nurses. In addition, prior to the pandemic, Sweden had small numbers of ICU beds and ICU nurses. Stress of Conscience also increases when working in the Covid-ICU compared to the working in the usual workplace. The most stressful aspect of conscience reported was that working in the COVID-ICU was so demanding, nurses did not have the energy to devote themselves to their family as they would have liked. This work: family conflict can be an explanation to earlier studies reporting that working in a COVID-ICU highly impacts the mental well-being of nurses.

## Data Availability

The datasets generated and analysed during the current study are not publicly available due to the participants confidentiality and the researchers have no ethical permission to share them. The corresponding author can be contacted if someone wants to request the data from this study.

## Abbreviations

ICU: Intensive Care Unit
ICPAN: International Collaboration of PeriAnaesthesia Nurses’
RN: Registered Nurses
SCQ: Stress of Conscience Questionnaire
